# Deep learning-based predictive identification of neural stem cell differentiation

**DOI:** 10.1038/s41467-021-22758-0

**Published:** 2021-05-10

**Authors:** Yanjing Zhu, Ruiqi Huang, Zhourui Wu, Simin Song, Liming Cheng, Rongrong Zhu

**Affiliations:** 1 Division of Spine, Department of Orthopedics, Tongji Hospital, Tongji University School of Medicine, School of Life Science and Technology, Tongji University, Shanghai, China; 2 Key Laboratory of Spine and Spinal Cord Injury Repair and Regeneration, Tongji University, Ministry of Education, Shanghai, China

**Keywords:** Computational neuroscience, Stem cells in the nervous system

## Abstract

The differentiation of neural stem cells (NSCs) into neurons is proposed to be critical in devising potential cell-based therapeutic strategies for central nervous system (CNS) diseases, however, the determination and prediction of differentiation is complex and not yet clearly established, especially at the early stage. We hypothesize that deep learning could extract minutiae from large-scale datasets, and present a deep neural network model for predictable reliable identification of NSCs fate. Remarkably, using only bright field images without artificial labelling, our model is surprisingly effective at identifying the differentiated cell types, even as early as 1 day of culture. Moreover, our approach showcases superior precision and robustness in designed independent test scenarios involving various inducers, including neurotrophins, hormones, small molecule compounds and even nanoparticles, suggesting excellent generalizability and applicability. We anticipate that our accurate and robust deep learning-based platform for NSCs differentiation identification will accelerate the progress of NSCs applications.

## Abstract (web summary)

The differentiation of neural stem cells (NSCs) into neurons is a critical part in devising potential cell-based therapeutic strategies for central nervous system diseases but NSCs fate determination and prediction is problematic. Here, the authors present a deep neural network model for predictable reliable identification of NSCs fate.

## Introduction

Stem cells have the capacity for self-renewal and differentiation, expanding the possible range of cell-based therapy in regenerative medicine; for example, potential applications may include restoring damaged neurons and recomposing tissue^1–3^. Neural stem cells (NSCs) have the potential to yield several cell types in the anatomy of the brain; therefore, strategies based on NSCs are considered valuable in the treatment of nervous system diseases, such as Alzheimer’s disease, stroke and traumatic brain injury^4–7^. NSCs are considered the “seed” cells of the central nervous system (CNS), capable of self-renewing and generating neurons and glia during CNS development. NSCs are tripotent cells that generate transit-amplifying progenitors and are later restricted to three lineages: neurons, astrocytes and oligodendrocytes^8–10^. Neurons are the essential units for information transmission in the nervous system, sending electrical and chemical signals to other cells through axons and receiving such signals via dendrites; neurons cannot reproduce or regenerate once they die^11^. Transplantation of exogenous NSCs or mobilization of endogenous NSCs, followed by their differentiation into neurons to reconstruct neural circuits damaged by neurological disorders, is a widely explored method for treating neurodegenerative diseases^12–16^. Astrocytes, the most abundant cell type in the CNS, communicate with and structurally support the growth of neurons, oligodendrocytes and endothelial cells, integrating into tripartite synapses and the neurovascular unit to perform their function. The signalling pathways of astrocytogenesis have attracted great interest as a means of suppressing the generation of astrocytes in many neurological disorders and generating a NSC model as a drug-screening platform^17,18^. In addition to participating in cellular communication, astrocytes also play an important role in neuroprotection by releasing neurotrophins, such as glial cell line-derived neurotrophic factor (GDNF), and reducing excitotoxicity in neurons^19^. Properly regulated astrocytes may provide neuroprotection, axonal guidance and vascular integrity after CNS injury^20^. Oligodendrocytes are myelinating glial cells that are important for neuronal electrical insulation, facilitating saltatory signal conduction^21^. Oligodendrocytes also provide the axons of neurons with metabolic and trophic support, including lactate, pyruvate and neurotrophic factors such as brain-derived neurotrophic factor (BDNF), via the myelin membrane^22^. Neurodegeneration can result in immune-mediated myelin sheath damage and subsequent axonal demyelination^23^. Researchers have made efforts to differentiate NSCs into oligodendrocytes that can contribute to postinjury remyelination, electrically insulating neuronal axons for impulse propagation and providing trophic and metabolic support for neurons^24–26^.

Therefore, the key mission in therapeutic applications is to efficiently induce NSCs into specific cell types, especially neurons. In this regard, several neurotrophins, small molecule drugs and hormones have proven to be effective, but researchers remain devoted to developing advanced approaches to directionally induce NSC differentiation^27–30^. Research progress in this area is limited by the fact that common laboratory methods to observe the effectiveness of inducing differentiation can be cumbersome, time consuming and uneconomical; examples include immunofluorescent staining, polymerase chain reaction (PCR) and western blots^31,32^. Determining the effect of potential inducers on NSC differentiation usually takes several days, and it can be affected by many factors, such as molecular marking techniques, immature laboratory technology, and the operational skill levels of experimental personnel. Sometimes, even limitations on the current understanding of essential biological characteristics of NSCs could become barriers to finding potential regulatory factors, since some mechanisms linked to NSC fate change, such as remodelling or dynamic changes in organelles, are poorly understood^33^. Current approaches might not be able to correctly identify the factors that act on these unclear mechanisms to affected NSC differentiation. Moreover, the current methods mentioned above are based on specific markers of each cell type, such as NeuN for neurons, GFAP for astrocytes and Olig2 for oligodendrocytes^34–38^. These markers are applicable only to specific cells, often those that are already at a certain stage of differentiation^39^. Thus, early identification of NSC differentiation is of great difficulty, limiting the development of related technical means. There is an urgent need for a more efficient, accurate, convenient and less wasting method; furthermore, it is better to have less influence on subjective judgement and less limit to the present understanding of neural development and differentiation.

Major advancements in biomedical applications of deep learning technology have occurred in recent years, including cell biology^40,41^. Studies have used deep learning to identify cell types, cell states and cell dynamic progress from flow cytometry or microscopy images^42–44^. Several recent reports have highlighted some noteworthy findings regarding the use of deep learning to observe and predict physiological processes in stem cells. One study showed that differentiation alters the morphology of haematopoietic stem cells. Deep learning is in a position to recognize these changes from microscopy data and predict the development of haematopoietic stem cells in advance by isolating cells earlier than the beginning of the known development progress^45^. Another study showed that machine learning could distinguish pluripotent stem cells from early differentiating cells^46^. These studies highlight the possible further application of deep learning in the field of stem cell therapy, and we speculate that machine learning could be used not only to identify the physiological variation among cells but also to judge the biological characteristics and changes caused by differentiation inducers. We hypothesize that NSCs could show similar features in a very early stage of differentiation under inducer treatment, and binding to specific receptors could lead to an initial effect of inducers on the cellular state, which might be extracted from high-throughput data by deep learning. Implementing the idea can help solve the existing experimental restricts of early identification of NSC differentiation and adjust NSCs-based treatment strategies, providing more practical applications of deep learning.

Using flow cytometry to detect cell markers is a common method with notable advantages, namely, high throughput, imageability and relatively high speed^47,48^. A cell-cycle study used imageable flow cytometry combined with machine learning to distinguish minor changes^49^, and a label-free imaging flow cytometry technique was developed based on coherent optical implementation of the photonic time stretch concept, achieving the advantages of high accuracy and fast speed^42^. The aforesaid findings emphasize that these two advanced approaches could be utilized to address the challenge of identifying NSC differentiation; specifically, imageable flow cytometry could provide large datasets to build deep learning networks.

**Fig. 1:**
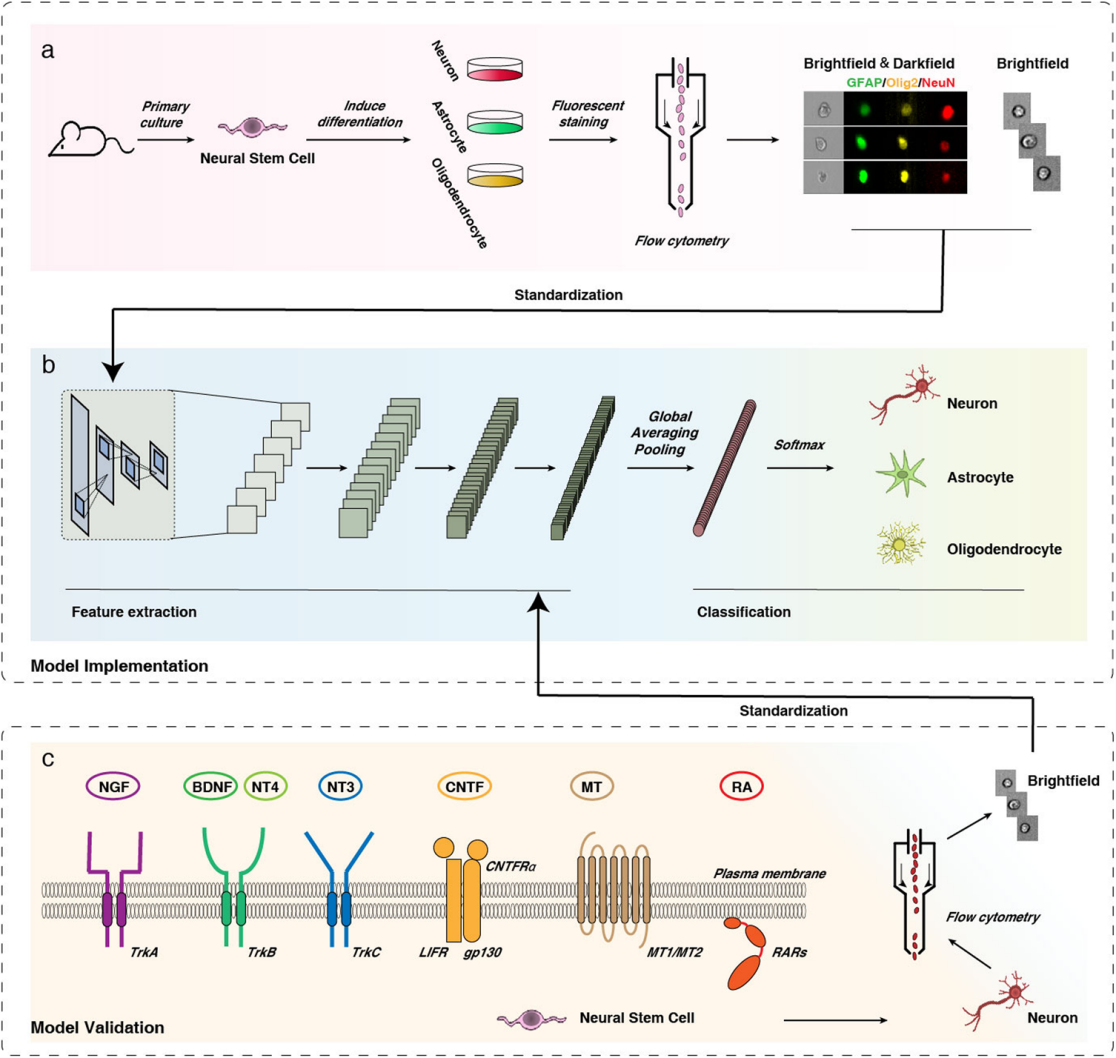
Overview of workflow. **a** NSCs were induced to differentiate into neurons/astrocytes/oligodendrocytes, and the cells were stained with NeuN (red)/GFAP (green)/Olig2 (yellow), collected and subjected to image flow cytometry. **b** Brightfield and darkfield (labelled) single-cell images were used as training data for the screening system; a schematic of the convolutional neural network (CNN) is presented. **c** Various inducers in different forms that act on different pathways were used to guide NSCs to differentiate into neurons, and brightfield (unlabelled) single-cell image patches were obtained by flow cytometry. These independent test sets were evaluated with the deep network model to show its generalizability.

Thus, we developed experiments to exploit the information in brightfield single-cell images for prospective identification of NSC differentiation. Referencing previous studies^50–56^, through our exploration and experiments, we produced different conditioned culture media to generate specific cell lines from NSCs (Fig. 1a). Several inducers with distinct forms and pathways were selected to test the idea and confirm the value of practical application of our model. Then, the cultured NSCs were marked and collected by imageable flow cytometry; in that way, we obtained 149,428 annotated single-cell images, among which 80% were used as training data to construct deep learning-based darkfield and brightfield models, and the other 20% were used for model testing. We used a convolutional neural network (CNN) to extract local image features, forming a well-architected image classification (Fig. 1b). Recent advances have shown that a CNN can use the convolution operation to establish local connections and to effectively build end-to-end data processing and recognition systems, which have been widely used in image processing^43,57,58^. CNNs combined with flow cytometry can achieve real-time separation of label-free individual target cells with high accuracy^59^. In order to further evaluate the general suitability of our system, 59,287 brightfield single-cell images for independent test data were obtained from independent experiments, which were performed using several common types of inducers, including neurotrophic factors, hormones and nanoparticles (Fig. 1c). In this way, we aimed to build a system that allows a CNN to recognize the features of differentiated NSCs via unlabelled brightfield single-cell images, which would provide a quick and simple approach to accurately identify NSC fate at an early phase, screen growth factors/small molecules/nanomedicines with high efficiency, and ultimately promote the medical application of NSCs.

## Results

### A deep learning-based model was established using single-cell images obtained from reliable differentiation experiments

To collect data for model construction, we developed a system that contained differentiating NSCs in different phases, generating neurons, astrocytes and oligodendrocytes and marking them using NeuN, GFAP and Olig2, respectively. To verify the differentiation efficiency of different groups with common laboratory methods, immunofluorescent staining, RT-PCR and western blot were employed. The detection time points were selected according to previously reported studies of differentiation into neurons (1, 3, 5 days), astrocytes (0.5, 1, 2 days), and oligodendrocytes (1, 2, 3 days), separately^60–62^.

**Fig. 2:**
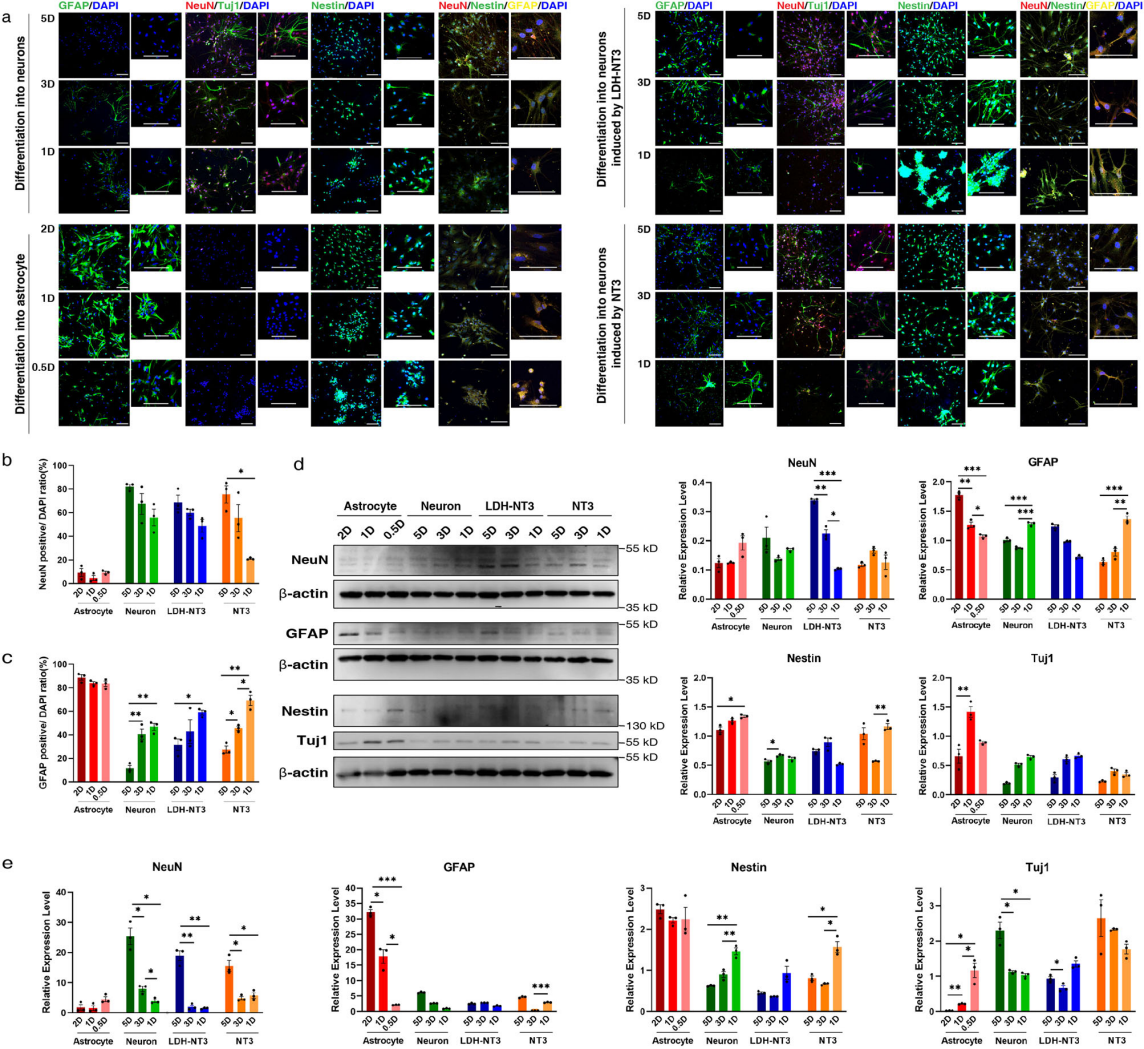
Differentiation efficiency of training and testing sets identified by immunofluorescence, western blot and RT-qPCR. **a** Images of immunofluorescent staining using NeuN, GFAP, Tuj1 and Nestin as characteristic markers of neuron and astrocyte differentiation induction at 5D (5 days), 3D (3 days), 2D (2 days), 1D (1 day) and 0.5D (0.5 days). Differentiated cells treated with NT3 and LDH-NT3 were stained following the same protocols. Quantification of immunostaining data. The *y-*axis shows the number of: **b** NeuN- and **c** GFAP-positive cells, *n* = 3 imaging field repeats. **d** Western blot analysis of NeuN, GFAP, Nestin and Tuj1 protein in NSCs in different states of differentiation, *n* = 3 biological repeats. **e** Quantitative real-time PCR detection of NeuN, GFAP, Nestin and Tuj1 gene expression in cells in different states of differentiation, *n* = 3 biological repeats. Data are shown as mean ± SEM. Statistical significance was determined by two-sided Welch’s ANOVA. ^*^p < 0.05, ^**^p < 0.01, ^***^p < 0.001. Scale bar = 100 μm. LDH: layered double hydroxide.

To verify the selected specific markers, we analysed their expression in NSC differentiation process as shown in Fig. 2a and Supplementary Figs. 3 and 4. In agreement with previous studies^35,60,63–65^, NSCs showed co-expression of GFAP and Nestin, while when treated with neuron differentiation medium, GFAP expression gradually decreased, and the proportion of NeuN+ cells gradually increased. By contrast, with astrocyte differentiation medium, the proportion of GFAP+ cells remained high, while that of NeuN+ cells remained low; over time, GFAP+ cells grew in size and exhibited morphological characteristics of astrocytes. Supplementary Fig. 4e shows that Olig2+ cells increase when treated with oligodendrocyte differentiation medium, while the number of GFAP+ cells is reduced. Results demonstrate that the NeuN, GFAP and Olig2 can clearly label the training data for deep-based model construction.

As shown in Fig. 2a–c, Supplementary Fig. 4e, f and Supplementary Table 1, under the influence of neuron differentiation medium with retinoic acid (RA) and sonic hedgehog (SHH), 82.073% of NSCs had differentiated into NeuN+ cells after 5 days of induction, 67.427% after 3 days and 55.827% after 1 day. Under the influence of differentiation medium with neurotrophin-3 (NT3), 75.407% of NSCs had differentiated into NeuN+ cells after 5 days of induction, 55.293% after 3 days and 20.997% after 1 day. With layered double hydroxide (LDH) loaded with NT3 nanoparticles (LDH-NT3), 68.823% of NSCs had differentiated into NeuN+ cells after 5 days of induction, 60.06% after 3 days and 48.73% after 1 day. Under the influence of astrocyte differentiation medium, 88.78% of NSCs had differentiated into GFAP+ cells after 2 days of induction, 83.813% after 1 day and 83.357% after 12 h. Under the influence of oligodendrocyte differentiation medium, 63.622% of NSCs had differentiated into Olig2+ cells after 3 days of induction, 55.472% after 2 days and 52.968% after 1 day.

The results of the western blot and RT-qPCR are shown in Fig. 2d, e. The expression of GFAP was highest in the astrocyte induction group and trended upward over time, while the expression of NeuN, Nestin and beta-III-tubulin (Tuj1) decreased over time. The expression of NeuN and Tuj1 was high in the neuron induction group containing RA and SHH, trending upward over time, while the expression of GFAP and Nestin decreased over time. Under the influence of LDH-NT3 and NT3, the expression of these markers had a trend similar to that of the neuron induction group, but weaker.

The above results indicate the reliability of setting differentiation conditions, as well as the clear labelling of the differentiation direction of NSCs. These differentiation methods were used along with flow cytometry, and single-cell images were collected to build and test darkfield and brightfield models. Immunofluorescent staining, RT-qPCR and western blot results demonstrated that the differentiated single-cell data were suitable for deep learning-based model training and testing.

**Fig. 3:**
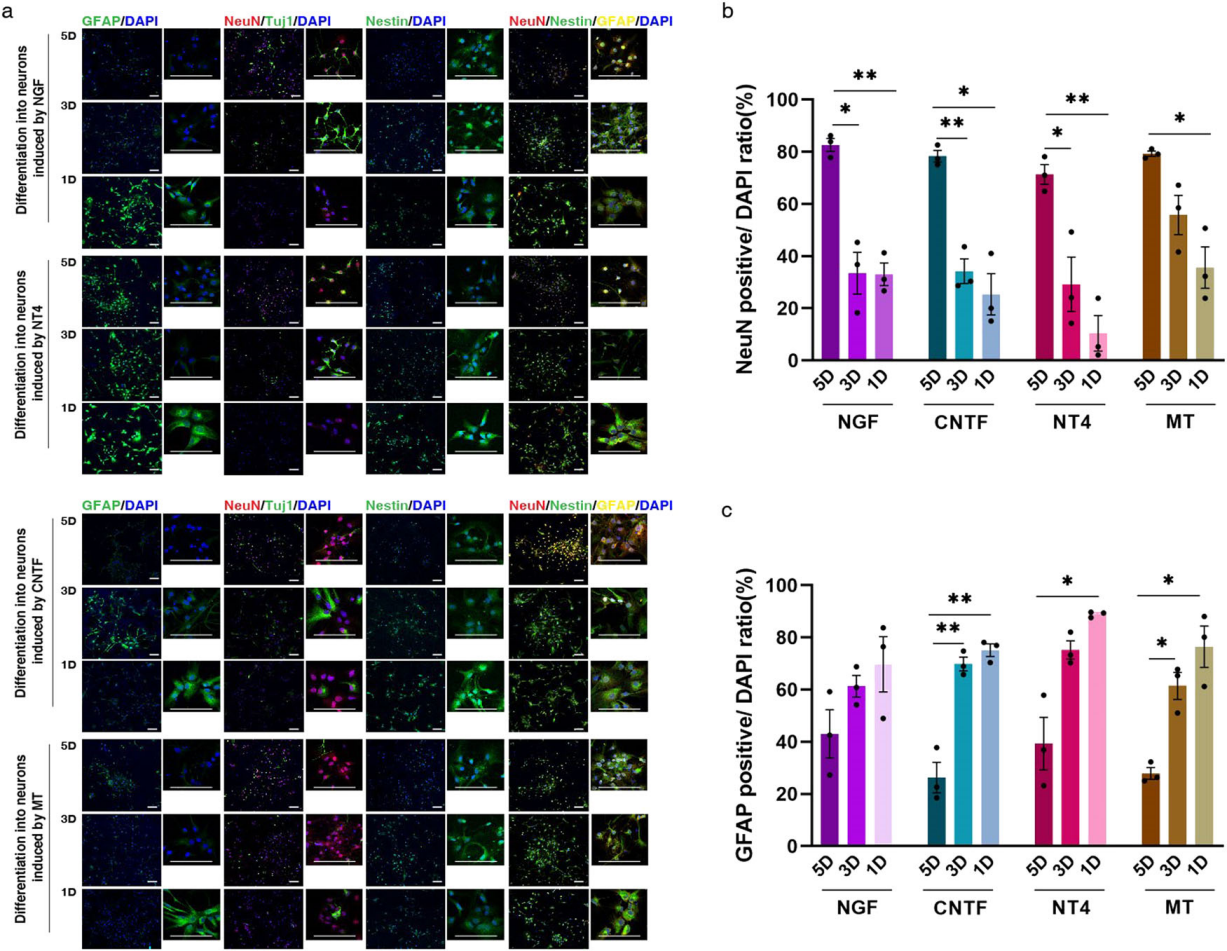
Differentiation efficiency of various neuron inducers, identified by immunofluorescence. **a** Images of immunofluorescence using NeuN, GFAP, Tuj1 and Nestin as characteristic markers of NGF/CNTF/NT4/MT-treated cells at 5D (5 days), 3D (3 days) and 1D (1 day). Quantification of immunostaining data. The *y-*axis shows the number of: **b** NeuN- and **c** GFAP-positive cells, *n* = 3 imaging field repeats. Data are shown as mean ± SEM. Statistical significance was determined by two-sided Welch’s ANOVA. ^*^p < 0.05, ^**^p < 0.01, ^***^p < 0.001. Scale bar = 100 μm.

For independent testing of the brightfield model, we obtained images of cells treated with various types of inducers. As shown in Fig. 3 and Supplementary Table 1, the immunofluorescence results show that, under the influence of neuron differentiation medium with neurotrophin-4 (NT4), 71.3333% of NSCs were differentiated into NeuN+ cells after 5 days of induction, 29.2333% after 3 days and 10.4333% after 1 day. Under the influence of neuron differentiation medium with nerve growth factor (NGF), 82.6333% of NSCs were differentiated into NeuN+ cells after 5 days of induction, 33.4667% after 3 days and 33.0333% after 1 day. Under the influence of neuron differentiation medium with melatonin (MT), 79.2667% of NSCs were differentiated into NeuN+ cells after 5 days of induction, 55.7667% after 3 days and 35.6333% after 1 day. Under the influence of neuron differentiation medium with ciliary neurotrophic factor (CNTF), 78.3333% of NSCs were differentiated into NeuN+ cells after 5 days of induction, 34.23% after 3 days and 25.3667% after 1 day. Immunofluorescence results demonstrated the efficiency of the chosen factors as inducers and the suitability of our dataset design.

### Deep learning prospectively identifies the differentiation of NSCs at a very early stage

We trained our model on the above-mentioned training set of 119,533 images and tested it on 29,895 images on both darkfield and brightfield channels. To efficiently leverage the dataset, we standardized all images to a size of 45 × 30, and our model achieved high performance on both mixed and independent test sets in terms of accuracy, receiver operating characteristic (ROC)-AUC and precision-recall (PR)-AUC, suggesting its advanced capacity to predict neural differentiation in different scenarios.

**Fig. 4:**
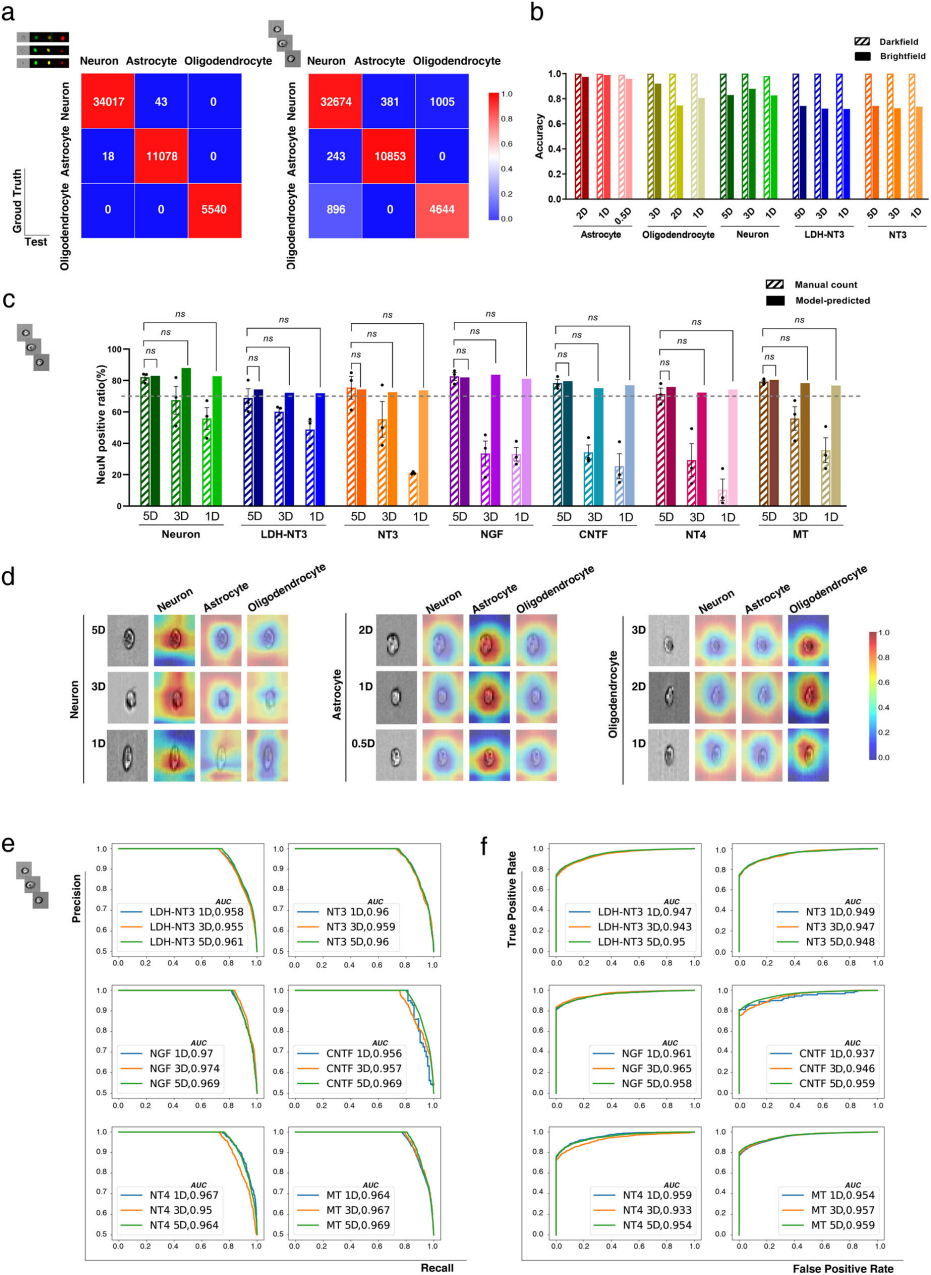
Deep learning prospectively identifies the differentiation of NSCs at 5D (5 days), 3D (3 days), 2D (2 days), 1D (1 day) and 0.5D (0.5 days). **a** Confusion matrices for the darkfield (left) and brightfield (right) models for the classification of each differentiated cell type. **b** Accuracy of each training set and the independent LDH-NT3 test set in both darkfield and brightfield models, the size of each testing dataset is available in Source Data file. **c** The proportion of neurons calculated by immunofluorescence and brightfield-based model benchmark, the size of each testing dataset is available in Source Data file. Data are shown as mean ± SEM, statistical significance was determined by two-sided single population *t*-test. ns: not significant. **d** The CAM highlights the class-specific discriminative regions of cells, blue represents low attention while red represents high attention. Benchmarking differentiated cell type predictions on independent testing data with: **e** ROC (receiver operating characteristic) and **f** PR (precision-recall) curves. LDH: layered double hydroxide.

As shown in Fig. 4a, b and Supplementary Table 2, the results suggest no significant difference in total accuracy between the fluorescently marked model (0.998) and the brightfield model (0.923), and the accuracy was high for each differentiation direction at each time point. It is especially interesting that not only the darkfield model but also the brightfield model achieved high accuracy in both mixed test groups (differentiated into neurons, astrocytes and oligodendrocytes; NT3-treated group) and the LDH-NT3-treated group, specifically, illustrating that the brightfield model has strong basic generalizability, which greatly streamlines the application process.

Surprisingly, as shown in Supplementary Table 1, when NSCs were guided to generate neurons, astrocytes and oligodendrocytes, the brightfield model precisely identified their final fate at 0.5–1 day with very high accuracy (82.72892% for the RA-treated set, 73.67773% for the NT3-treated set, 71.89459% for the LDH-NT3-treated set, 95.86375% for the astrocyte group and 80.59837% for the oligodendrocyte group), consistent with the 5 day experimental observations, whereas common approaches merely demonstrate the instantaneous differentiation status at specific time points by immunostaining. The results indicate that our model can use unlabelled single-cell brightfield images to predict NSC fate within 1 day, which makes it a very valuable approach for NSC research, not only to increase efficiency and reduce cost in the area of molecular screening but also to suppress interference caused by statistical errors, and markers such as GFAP, which expressed in both early differentiated NSCs and astrocytes.

In order to further evaluate the generalization of our system to reliably predict putative NSC differentiation direction in independent experiments that used NGF, NT4, CNTF, MT and LDH-NT3 as inducers for producing neurons, we benchmarked our model on 59,287 datasets with the metrics of accuracy, ROC-AUC and PR-AUC. On all datasets, as shown in Fig. 4c, Supplementary Table 1 and Supplementary Table 2, the brightfield-based model achieves excellent prediction of neuron proportions at 1 day (81.09294% on the NGF set, 74.197% on the NT4 set, 76.92308% on the CNTF set, 76.79027% on the MT set and 71.89459% on the LDH-NT3 set), which is closely consistent with the 5 days immunofluorescence counts. However, common laboratory methods could not identify differentiation at very early stages at 1 day and 3 days, asserting the superiority of our model over existing approaches. Furthermore, the system gained records on both ROC-AUC and PR-AUC, as shown in Fig. 4e, f, suggesting excellent robustness and capability to minimize the imbalance and false positives.

### Class activation maps offer insight into the functional mechanisms of medications

To understand how our model interprets the input cell images, we introduced class activation mapping techniques^66^. The input cell images shown in Fig. 4d are randomly selected from each treated group. Given these images, the activation maps of each layer block were extracted. Of these maps, Supplementary Fig. 5 presents six examples per block due to space limitations, although each layer block has hundreds of activation maps. The layer block-wise activation maps depict the appearance of the input images or the activation maps from the previous layer block processed in units of the current layer block. Moreover, we established the final class activation maps (Fig. 4d). These class activation maps are used to describe the model’s receptive field in the case of given classes, where blue represents low attention while red represents high attention. Results show the attention of the model is concentrated on the cell, including its edges and internal parts, when the given class matches the predicted class, and distracted otherwise, indicating that the model tends to perform its classification based on the details of both edges and internal parts of the cells.

### Performance comparison with other neural network architectures

**Fig. 5:**
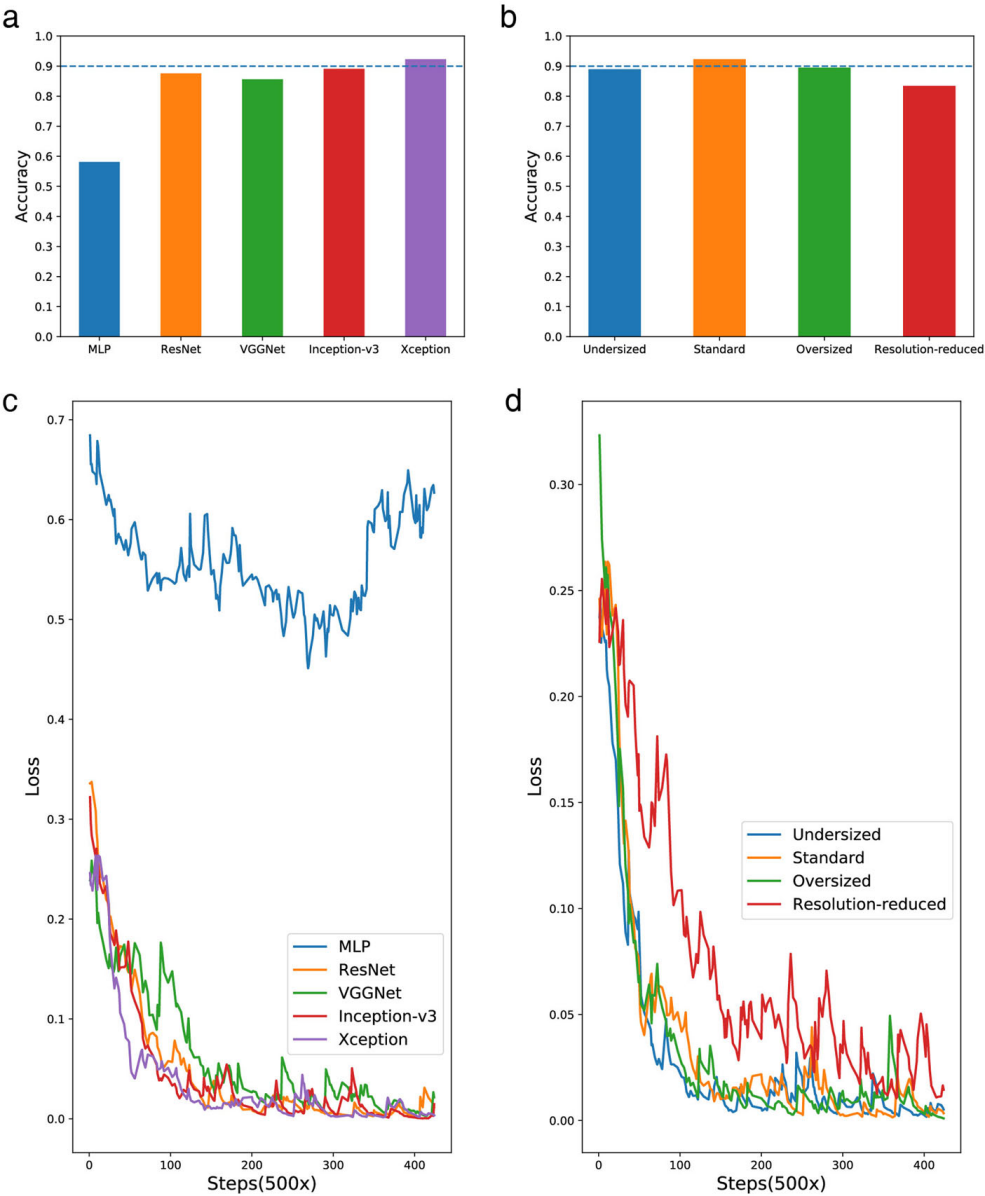
Performance comparison of models established with different structures. Accuracy comparison of models with: **a** different architectures and **b** Xception models with different size and input resolutions. Loss curves comparison of models with: **c** different architectures and **d** Xception models with different size and input resolutions. The size of each testing dataset is available in Source Data file. MLP, multi-layer perceptron.

We compared the performance of our Xception-based model^67^ with models established using other deep learning techniques, including multi-layer perceptron, ResNet^68^, VGGNet^69^ and Inception-v3^70^ architectures. These trained models utilize the same training and testing dataset (containing 119,533 images and 29,895 images, respectively) as the Xception-based model above. Figure 5a compares the accuracy of each model on the testing dataset, showing that the Xception-based model is the only one that exceeds 0.9. Additionally, the loss curves of the compared models are depicted in Fig. 5c, showing that the curve of the Xception-based model lowers quickly in the early steps and stabilizes at nearly the smallest loss value among all the network architectures, representing good robustness. These comparisons indicate that the chosen Xception method performs the best among the five deep learning techniques.

Moreover, we built and evaluated three additional Xception-based models, including an undersized version, an oversized version and a resolution-reduced version, with the same training and testing datasets, where the undersized version was 30% smaller than the standard version while the oversized version was 30% larger. For the resolution-reduced version, the size of the input images was reduced to 15 × 10, in contrast to the standard version, whose input images were 45 × 30. Figure 5b depicts a comparison of accuracy among the four models, showing that the standard version remained the highest. Regarding training robustness, the standard version gave the best performance once again, as shown by the comparison of loss curves in Fig. 5d. The comparison in this part indicates that the undersized version and oversized version, respectively, underfit and overfit the data, in contrast to the standard version of the model; additionally, shrinkage of the input images caused a shortage of effective information, making the corresponding model perform poorly.

## Discussion

Transplantation of NSCs offers exciting possibilities for CNS regeneration, but it is challenging to guide the differentiation of NSCs into specific cell types. Biomarkers are commonly used to examine differentiation towards distinct cell fates, relying on the characteristics of cells during the neurogenesis process^71^. Despite a variety of studies on the factors regulating the fate of NSCs, the neurogenesis process remains to be fully elucidated, especially the cellular changes at the very early stage of differentiation into neurons^72,73^; thus, it is difficult to identify the direction of differentiation early in the process. An identification process must be generalizable to support the advanced development of efficient agents for neurodegenerative diseases and neurological injuries: it needs to apply to any effective substance, independent of what its pathways may happen to be. Advanced instrumentation facilitates data collection, but one of the most challenging obstacles is that the data are often uninterpretable within the limitations of current devices. Existing approaches are based primarily on human cognition, but differentiating subtle cellular morphologic changes or predicting drug interactions is challenging for human beings^74–76^. Deep learning enables automatic feature extraction utilizing vast datasets to offer solutions to perplexing problems, including those encountered in the biomedical field^77,78^. Thus, we decided to take full advantage of abundant single-cell images by using deep learning to prospectively identify the differentiation of NSCs.

First, we innovatively devised and framed a very accurately marked NSC differentiation dataset, ensuring the effectiveness of model training. The NSC fate identification system was built based on the confirmed experimental data, and it is critical to successfully extract the characteristic information of each cell type. An elegant CNN structure produced a highly efficient and strongly generalizable model. We then tested the performance of our system, and the results suggested that our model could estimate the proportion of the final differentiated cell type in the early stages of differentiation, before common laboratory techniques could detect the corresponding alterations.

When gathering the cell images, the repeatability of experiments matters to the further application for the model, thus the cell status for training data is important. The proliferation and differentiation abilities of prolonged cultured NSCs of late passages have differences with early passages, and p0–p2 cells from primary culture may not be able to form enough amount of neurospheres with stable status for collecting high-throughput data, thus researches usually take NSCs from p3 to p9^79–84^. We conducted experiments to evaluate the state of p3–p5 NSCs, results show strong expression of specific markers Nestin and PAX6 in all groups (Supplementary Fig. 2a), indicating certain proliferation capacity. Immunostaining results of measuring differentiation ratio of p3–p5 NSCs demonstrated the consistent differentiation ability (Supplementary Fig. 2b, c), indicating that NSCs at p3–p5 are suitable for model building data collection. Regarding our training data, RA induces NSCs to differentiate into neurons by binding to RA receptors (RARs)^85^, whereas the combination of thyroid hormone (T3) and platelet-derived growth factor (PDGF) promotes the differentiation of oligodendrocytes through the Wnt/β-catenin signalling pathway^86^, CNTF, with high concentrations acting via the JAK-STAT signalling pathway to differentiate cells into astrocytes^87^. Furthermore, we compiled independent testing data on NSC differentiation using a variety of neuronal inducers targeting different receptors (Fig. 1c). First, we used several neurotrophins, including NGF, BDNF, NT4 and NT3, to regulate NSC differentiation into neurons. Neurotrophins are peptide growth factors implicated in neuronal differentiation; they bind to their related receptor tyrosine kinases (Trks), which are located in the plasma membrane of responsive cells. NGF preferentially binds to TrkA, BDNF and NT4 preferentially bind TrkB, and NT3 acts on TrkC^88,89^. Next, low-concentration CNTF was used as another neuronal inducer. This inducer, which belongs to the haematopoietic cytokine superfamily, acts by binding to CNTF receptor α (CNTFRα) and gp130, ultimately recruiting leukaemia inhibitory factor receptor β (LIFRβ) and activating the mitogen-activated protein kinase pathways^87,90^. Subsequently, MT was utilized to facilitate neuron differentiation mediated via activation of MT receptors 1 and 2 (MT1 and MT2)^91^. Moreover, recent studies on the impact of nanoparticles on NSC fate were taken into consideration^92,93^. LDH has been shown to assist NT3 in regulating stem cell fate^94,95^; to determine the general validity of our system, we tested it with LDH-NT3 as an unconventional type of inducer with an unclarified mechanism.

Additionally, we performed a study to minimize the duration of inducer treatment in order to improve the efficiency of model training and application; in that study, different time points were carefully selected for cell harvesting. Research indicates that when machine learning determines very early whether embryonic stem cells differentiate or not, its judgements may be based on cell-level variations in biological processes^46^, suggesting that the physiological process of NSC differentiation is the critical factor that needs to be considered when set the time points. Previously, studies show that it takes different differentiation time when NSCs covert into each lineage, around 7 days for neurons^60^, 3 days for astrocytes^61^ and 5 days for oligodendrocytes^62^, respectively. At these time points, cells start to appear their specific markers expression and functional properties. Our greatest concern is to identify NSC fate in the early stage of differentiation and determine the actions of the inducers, thus shorter time points are set to establish the predictable model. We tested the gene expression levels to observe the changes in NSCs under the action of different inducers to design further data collection procedures for model construction. As the results show in Supplementary Fig. 4, for cells undergoing neuronal differentiation, the expression level of NeuN firstly showed a significant increase at 1 day, between 3 days and 1 day there is an observable difference and at 5 days also there appears a significant increase, while there are no significant changes at earlier time points of 0, 2, 4, 8 and 12 h; thus, 1 day was set as the earliest time point for detection. For astroglial and oligodendroglial differentiation, obvious changes in GFAP and Olig2 appeared at 2 days and 3 days, and significant changes first appeared at 12 h and 1 day, respectively. Therefore, the earliest expected time points for effective prediction by our prediction system were set to 12 h for astrocytes and 1 day for neurons and oligodendrocytes. The results showed that, after these minimum durations, our model could predict the differentiation direction and ratio of cells several days in advance, which may be due to its sensitivity to subtle changes in cell state or organelle morphosis.

In our previous work, we built an Inception 3-based neural network that could analyse the distinction of apoptotic cells; however, we noticed that the complicated structures and numerous manually defined branches increased the difficulty of training, which might affect the efficiency of the optimal model, and the results showed that its performance in brightfield detection had room for improvement^96^. In the current study, we used a better-constructed neural network based on Xception, which reduced the complexity of the model and made it more concise and clear, and this approach proved to be very effective. The present network builds the optimization model from simple training with the hyperparameters of basic training parameters and default learning rate; in particular, unlabelled brightfield images can fully meet the modelling requirements with excellent accuracy of 0.923. The results of both mixed testing data and multiple independent experiments demonstrated that the mechanism does not influence the effect of the model’s verdict, nor does the form of inducer (Fig. 4a and Supplementary Tables 1 and 2). Our system is able to prospectively identify the direction of NSC differentiation in 1 day using non-fluorescently labelled cell images, and the ROC-AUC/PR-AUC verified its high robustness and precision (Fig. 4e, f and Supplementary Tables 1 and 2). Class activation mapping, shown in Fig. 4d, suggested that our model can identify very small morphological variations in cellular structures; thus, it was able to classify cell fate by discriminating differences between each type, which is the main strength of machine learning. Furthermore, we explored the predictive performance achieved by CNN architectures with various layer structures, branching strategies, network capacities and resolutions of input cell images. The results of the performance comparison on the test set demonstrated the superiority of the architectures that our model adopts.

In addition to the above advantages, our model has the potential for functional expansion to cover a variety of applications; for instance, predicting neuronal subtypes such as motor neurons or dopaminergic neurons would be a very meaningful development. Dopamine neuron transplantation can innervate the striatum and improve motor asymmetry and is a promising therapy for Parkinson’s disease^97^. Transplantation of motor neurons derived from stem cells can significantly promote motor function recovery in amyotrophic lateral sclerosis^98^. The development of a more efficient identification system for specific neuron populations will undoubtedly promote cell-based therapy for specific neurodegenerative diseases. At present, however, there are a few hurdles to overcome before this further application. First, the differentiation ratio of NSCs or induced pluripotent stem cells into a specific population is not sufficient to gather a suitable dataset to train the model. According to a previously reported study, under 40% of stem cells treated with several cytokines can differentiate into dopamine neurons or motor neurons^64,99–102^. In order to build a training set for machine learning, correct classification and labelling are necessary. In addition to isolating the specific subpopulations, flow cytometry/cell sorting should be used to enrich the target cells, but the cells have been secondarily treated before analysis, which introduces uncontrolled variables and reduces training efficiency. Meanwhile, specific markers for neuron subpopulation profiling and selection remain under investigation^60^, making it difficult to clearly tag data. Another valuable possible extension of the model is to explore the region heterogeneity of NSC differentiation. Heterogeneity describes the regionalization of NSC niches, progenitor cells in different regions express different genes to become different subtypes of cells, appears to be a key feature of NSC development in the CNS, but how it affects NSC fate and function is not fully understood^35,103^. It is worth considering isolating regional NSC subpopulations, taking advantage of the model to effectively identify subtle cellular variation during the differentiation progress and even explore the impact of different inducers on NSC subtypes development. Some limitations need to be addressed before further exploration, first, to resolve additional layers of heterogeneity, optimized protocols are needed to be established for precise isolation and effective enrichment of regional subtypes.^104^ Additionally, research shows that existing exploration of regional heterogeneous NSC subtypes is to observe the changes of gene expression using single-cell RNA-sequencing, however, there is still lack of recognized specific markers to identify cells from different regions including ventricular-subventricular zone (V-SVZ) or subgranular zone (SGZ). Besides, lineage-tracing studies indicate that regional specification is established very early in embryonic development, but the progress of its development still needs to be more clearly elucidated in order to set up suitable time points for collecting the regional NSC subtypes^105^. Though there are difficulties, we believe the extension to the deep-based model application is well worth the efforts due to its strong applied value, suggesting that our system can be used not only in predicting NSC fate and screening neural induction factors but also in research on NSC reactivation and development.

In summary, we provide an advanced tool for the identification of NSC fate; this model has the key features of high precision, a low false positive rate, speed, wide applicability, simple operation and cost-effectiveness. After NSCs are co-cultured for 1 day with the inducer to be studied, our system can offer a predictive differentiation result with a single-cell image. This could be a convenient and well-suited method to investigate the unknown effects of substances on NSCs and rapidly predict potential therapeutic molecules and drugs to promote neural regeneration in CNS diseases. We are also working with further extensions and applications of our system, attempting to develop a multifunctional approach for NSC research.

## Methods

### Experiments and data collection

#### Primary NSC processing and culture

Primary NSCs were processed as follows: Embryonic SD rats (E13.5) were chosen for cell extraction. The brain of each embryo was isolated under a stereoscopic microscope, washed with 1× phosphate buffer saline (PBS, Gibco, catalogue no. 10010023) and then incubated with 0.125% trypsin solution (Gibco, catalogue no. 25200056) at 37 °C for 3 min. Next, DMEM containing 10% foetal bovine serum (FBS, Gibco, catalogue no. 040011 A) was applied to neutralize the trypsin activity. The suspension was filtered with 70 μm mesh cell strainers (BD Biosciences, catalogue no. 087712). The filtered cell suspension was centrifuged and resuspended in DMEM/F12 (Gibco, catalogue no. 11320033) containing 2% B27 (Gibco, catalogue no. 17504044), 1% N2 (Gibco, catalogue no. 17502001), 20 ng/ml basic fibroblast growth factor (bFGF, Peprotech, catalogue no. 45033) and 20 ng/ml epidermal growth factor (EGF, Peprotech, catalogue no. 31509). Primary NSCs were cultured in proliferation medium for 2 days until neurosphere formation. The neurospheres were centrifuged and incubated with Accutase (Stem Cell, catalogue no. 7920) for 5 min in a 37 °C incubator to obtain single NSCs for continuous passage culture. NSCs were passaged every 4 days with Accutase after neurospheres formed, and half of the culture solution was refreshed every 2 days. Animals were obtained from Shanghai Slac Laboratory Animal Co., Ltd., and raised in the SPF animal laboratory at Tongji University. All protocols were approved by the Institute of Laboratory Animal Resources of Tongji University and complied with the Guide for the Care and Use of Laboratory Animals of the National Institutes of Health. Ethical and legal approval for this study was obtained from the Institute of Laboratory Animal Resources Animal Care and Use Committee. All efforts were made to minimize animal suffering and sacrifice.

#### NSC identification and differentiation

NSCs at p3, p4, p5 were identified by immunostaining, using specific markers Nestin and PAX6. For inducing differentiation experiments, cells were seeded onto poly-L-ornithine-coated 6-well plates, and after 12 h of incubation at 37 °C, the proliferation medium was replaced by differentiation medium. The composition of the astrocyte differentiation medium was as follows: DMEM (Gibco, catalogue no. 11965092), 10% FBS, 20 ng/ml CNTF (Peprotech, catalogue no. 45013), 10 ng/ml EGF and 10 ng/ml bFGF. The composition of the oligodendrocyte differentiation medium was as follows: DMEM/F12, 10 ng/ml EGF, 20 ng/ml PDGF (Peprotech, catalogue no. 10014B) and 50 nM T3 (ProSpec-Tany, catalogue no. hor001). The composition of the neuron differentiation medium with RA (Sigma, catalogue no. R2625) and SHH (Peprotech, catalogue no. 31522) was as follows: Neurobasal (Gibco, catalogue no. 10888022), 2% B27, 1% N2, 10 ng/ml BDNF (Peprotech, catalogue no. 45002), 50 ng/ml insulin-like growth factor (IGF, Peprotech, catalogue no. 10011), 0.1 μM c-AMP (Sigma, catalogue no. A9501), 1 μM ascorbic acid (AA, catalogue no. A4403), 1 μM RA and 1 μM SHH. The composition of the neuron differentiation medium with NT4 (Peprotech, catalogue no. 45004) was as follows: Neurobasal, 2% B27, 1% N2, 10 ng/ml BDNF, 50 ng/ml IGF, 0.1 μM c-AMP, 1 μM AA and 20 ng/ml NT4. The composition of the neuron differentiation medium with NGF was as follows: Neurobasal, 2% B27, 1% N2, 10 ng/ml BDNF, 50 ng/ml IGF, 0.1 μM c-AMP, 1 μM AA and 10 ng/ml β-NGF (Peprotech, catalogue no. 45001). The composition of the neuron differentiation medium with CNTF was as follows: Neurobasal, 2% B27, 1% N2, 10 ng/ml BDNF, 50 ng/ml IGF, 0.1 μM c-AMP, 1 μM AA and 100 ng/ml CNTF. The composition of the neuron differentiation medium with MT (Sigma, catalogue no. M5250) was as follows: Neurobasal, 2% B27, 1% N2, 10 ng/ml BDNF, 50 ng/ml IGF, 0.1 μM c-AMP, 1 μM AA and 25 mM MT. The composition of the differentiation medium with NT3 (Peprotech, catalogue no. 45003) was as follows: Neurobasal, 2% B27, 1% N2, 10 ng/ml BDNF, 50 ng/ml IGF, 0.1 μM c-AMP, 1 μM AA and 20 ng/ml NT3. The composition of the differentiation medium with LDH-NT3 was as follows: Neurobasal, 2% B27, 1% N2, 10 ng/ml BDNF, 50 ng/ml IGF, 0.1 μM c-AMP, 1 μM AA, LDH-NT3 with 0.08 ng/ml NT3, and 2 μg/ml LDH.

#### LDH-NT3 preparation and characterization

LDH was prepared as follows: 0.544 g NaOH was dissolved in 80 ml deionized water (ddH_2_O) under N_2_ atmosphere, then a mixture of 1.538 g Mg(NO_3_)_2_·6H_2_O and 0.75 g Al(NO_3_)_3_·9H_2_O was dissolved in 20 ml ddH_2_O, and added dropwise in the NaOH solution. After 30 min stirring at 500 rpm, the product was washed thrice with ddH_2_O, and dispersed in ddH_2_O. After keeping for 16 h hydrothermally at 100 °C, LDH was obtained by centrifugation. NT3 was loaded into LDH using ion exchange intercalation. The morphological features were observed by transmission electron microscopy (TEM). LDH and LDH-NT3 have a size of approximately 100 nm, with a hexagonal lamellar structure (Supplementary Fig. 1).

#### Flow cytometry

NSCs treated with differentiation medium were collected with Accutase at different stages and washed thrice with PBS before being fixed. The cells were fixed in cold 80% methanol for 5 min and then disrupted using 0.1% Triton X-100 on ice for 20 min. Later, the cells were incubated with anti-GFAP (Bioss, catalogue no. bs-0199R-AF488, 1:200), anti-NeuN (Bioss, catalogue no. bs-10394R-APC, 1:200) and anti-Olig2 (Bioss, catalogue no. bs-11194R-PE, 1:200) antibodies for 2 h on ice. The cells were washed three times with PBS before flow cytometry. Eventually, approximately 20,000 stained cells were collected and photographed. Experiments of the training sets (astrocyte, neuron, oligodendrocyte and NT3-treated groups) were carried out in the Key Laboratory of Spine and Spinal Cord Injury Repair and Regeneration of the Ministry of Education; Experiments of independent test for the darkfield model (LDH-NT3-treated group) were carried out in the laboratory of the School of Life Science and Technology of Tongji University. Images of the training and the darkfield model independent test groups were gathered using a FlowSight apparatus (Merck Millipore) at Tongji University. The brightfield model independent test data (NT4-, NGF-, CNTF-, MT-treated groups) were collected in the laboratory of Tongji Hospital and photographed by ImageStream Mark II apparatus (Merck Millipore) at the Chinese Academy of Sciences Shanghai Institute of Materia Medica. Single-cell images were gained from IDES v6.1. Each laboratory has its own experimenters to conduct the experiments.

#### Immunofluorescence

Immunofluorescence assays were performed as follows: the cells were washed three times with PBS and fixed with 4% paraformaldehyde for 15 min. Next, the cells were washed and blocked with PBS containing 5% donkey serum and 0.3% Triton X-100 at 37 °C for 1 h, then incubated with primary antibodies at a suitable dilution ratio in blocking solution at 4 °C overnight. Afterward, the cells were washed to remove residual antibody. The secondary antibody was applied at a suitable dilution ratio at 37 °C for 1 h. 4′,6-Diamidino-2-phenylindole (DAPI) was used to stain the nuclei. The antibodies used were as follows: anti-NeuN (Abcam, catalogue no. ab190195, conjugated with Alexa Fluor 488, 1:200), NeuN (Abcam, catalogue no. ab104225, 1:500), anti-NeuN (Millipore, catalogue no. MAB 377, 1:100), anti-GFAP (Abcam, catalogue no. ab53554, 1:1000), anti-Tuj1 (Abcam, catalogue no. ab78078, 1:1000), anti-Nestin (Abcam, catalogue no. ab6320, 1:500), anti-PAX6 (Abcam, catalogue no. ab195045, 1:350), anti-Olig2 (Abcam, catalogue no. ab109186, 1:100), rabbit anti-mouse IgG (YEASEN, catalogue no. 33912ES60, 1:200), donkey anti-mouse IgG (Abcam, catalogue no. ab150105, 1:1000), donkey anti-goat IgG (Abcam, catalogue no. ab175704, 1:1000) and donkey anti-rabbit IgG (Abcam, catalogue no. ab150075, 1:1000). DAPI (Sigma, catalogue no. D9542) was used to stain the nuclei. A Zeiss confocal microscope (LSM 700, Carl Zeiss, Jena, Germany) was applied to observe the fluorescence signal. Image acquisition was done with ZEN 2.3 (blue edition, Carl Zeiss) and micrographs were assembled using Adobe Illustrator CC 2018.

#### Western blot assay

The total protein content was extracted from cells using nucleoprotein and cytoplasmic protein extraction kit (KeyGen, catalogue no. KGP250) according to the manufacturer’s instructions. Then, a western blot assay was performed as follows: protein was mixed with 5× protein-loading buffer (KeyGen Biotech., Nanjing, China) and boiled at 95 °C for 5 min before being separated on a 12% SDS-PAGE gel. After electrophoresis, proteins were transferred to PVDF membranes (EMD Millipore, Billerica, MA, USA) by applying a current of 300 mA for 100 min in an ice bath. Then, the membranes were blocked with 5% BSA solution containing 0.1% Tween-20 (TBST) for 30–60 min at room temperature. The blocked PVDF membranes were incubated with primary antibodies at 4 °C overnight and washed three times with TBST. The antibodies used were as follows: anti-NeuN (Abcam, catalogue no. ab104225, 1:5000), anti-Nestin (Abcam, catalogue no. ab6320, 1:1000), anti-GFAP (Abcam, catalogue no. ab53554, 1:10,000), anti-Tuj1 (Abcam, catalogue no. ab78078, 1:1000), anti-β-actin (ABWAYS, catalogue no. AB0035, 1:5000), goat anti-mouse HRP (Abcam, catalogue no. ab205719, 1:5000) and goat anti-rabbit HRP (Abcam, catalogue no. ab205718, 1:5000). Blots were visualized with an ImageQuant LAS 4000 mini (GE Healthcare Life Science). Densitometry of protein bands was measured with ImageJ 1.47v and normalized to the respective internal control (β-actin) band. Graphs and statistical analysis were done in GraphPad Prism 8 version 8.4.2.

#### Quantitative real-time reverse transcription polymerase chain reaction (RT-qPCR)

The total RNA of cells was isolated with RNAiso plus (Takara, catalogue no. 9108). The concentration and purity of RNA samples were measured with a Nanodrop ND-2000 (Thermo Science, MA, USA). cDNA was synthesized with a Primer Script Reverse Transcriptase Kit (Takara, catalogue no. RR037A). Quantitative real-time PCR was performed using a TB Green^TM^ Premix Ex Taq Kit (Takara, catalogue no. RR820A) on a LightCycler Real-Time PCR System (Roche, 480II). The primer sequences (Sangon Biotech) are listed in Supplementary Table 3. The relative amounts of mRNA were calculated using the ΔΔCt relative quantification method. Glyceraldehyde-3-phosphate dehydrogenase (GAPDH) served as the control gene, and the mRNA levels of specific genes were normalized to GAPDH. Calculations and statistics were done in Microsoft Excel version 16.36; graphs were plotted in GraphPad Prism 8 version 8.4.3.

### Image data preprocessing

The cell images were preprocessed before feed into the neural network. The processing details are listed below: first, resize each image to 45 × 30 with OpenCV package; second, divide each resized image pixel value by 255 to squash the value domain down to 0–1.

### Deep learning techniques

#### Convolutional neural network for classification

We implemented a set of CNNs to perform the classification task for each cell image.

These CNNs were constructed with the “Xception” module, which is an upgraded version of the “Google Inception” module. The difference between the “Google Inception” module and a common linearly connected convolutional layer aggregate is that the former adopts a “network-in-network” structure containing branches to extract further useful information from the data flow. The number of branches between two specific layers is usually 2 or 3. Compared with the “Google Inception” module, the “Xception” module uses an extreme policy to perform one spatial convolution for every output channel of the 1 × 1 convoluted input data. With this policy, the “Xception” network surpasses the “Google Inception” network in various benchmarks. In addition, the “Xception” module is much simpler and more compact than the “Google Inception” module, making the whole network simple, clear and highly extendable.

#### Model training methodology

We used the Adam optimizer combining the momentum and exponentially weighted moving average gradients methods to update the weights of the networks.

The networks were trained with the PyTorch framework on four NVIDIA GTX 1080Ti GPUs. The parameters of the Adam optimizer are as follows: learning rate of 0.001, beta1 of 0.9, beta2 of 0.999 and epsilon of 10^−8^.

#### Activation mapping for each layer block

First, we randomly selected a total of 9 cell images, with each treated group providing one image. Given these cell images, the activation maps of each layer block of the model were collected. Due to space limitations, only six activation maps per layer block per image are presented in Supplementary Fig. 5.

#### Class activation mapping for feature identification

We adopted the class activation mapping technique to generate a comprehensive view with which to understand the recognition paradigm of the networks. For this method, the last three layers were as follows: a feature convolution layer, a global average pooling layer and a 1 × 1 convolution layer without an activation function. In order to produce the class activation map, the weights of the final 1 × 1 feature convolution layer were extracted. Then, the selected input images, which were the same as the input images used by the layer block-wise activation mapping process, were used to perform the forward pass, collecting the feature maps of the feature convolution layer. Finally, the class activation maps were calculated by the weights on the linear combinations of these feature maps, and images were generated through the Python visualization package “Matplotlib” (3.2.2 version).

#### Other related neural network architectures

We implemented various other types of neural networks to compare with our Xception-based network, including multi-layer perceptron, ResNet, VGGNet and Inception-v3 networks.

The multi-layer perceptron had three layers, namely, the input layer, the hidden layer and the output layer. The input layer took a 45 × 30 image as input and transformed it into a vector of length 512, which was fed into the 512 × 512 hidden layer. Finally, the output layer used the output of the hidden layer to perform the three-way classification.

For the ResNet, VGGNet and Inception-v3 networks, we used the classic layer configuration of each network with customized starting layers to fit the size of the cell images.

### Statistics and reproducibility

All values are presented as mean ± SEM calculated by GraphPad Prism 8 version 8.4.3. Two-sided Welch’s ANOVA was used to identify significant differences between different groups in immunofluorescence, RT-qPCR and western blot experiments in Figs. 2 and 3. Two-sided single population *t*-test was applied to identify significant differences between the proportion of neurons calculated by immunofluorescence and brightfield-based model benchmark in Fig. 4c. *p* < 0.05 was taken to indicate a statistically significant difference. Statistical significance levels are denoted as follows: ^*^p < 0.05; ^**^p < 0.01; ^***^p < 0.001. Results of Shapiro-Wilk normality test are shown in Supplementary Fig. 6. *p* > 0.05 was taken as for passing the normality test. In general, *n* values refer to the number of biological repeats or imaging fields for a given experiment; details are provided for each experiment in the corresponding figure legend. All data from representative experiments were repeated three times independently with similar results.

We tested the performance of our model on independent datasets using metrics of accuracy, ROC-AUC and PR-AUC, as well as generated the CAM figures given the exemplary cell image without retraining the networks. All attempts at replication were successful with similar results.

### Reporting summary

Further information on research design is available in the Nature Research Reporting Summary linked to this article.

## Supplementary information

Supplementary Information

Reporting Summary

## Data Availability

The main data supporting the findings of this study are available within the article and in the Supplementary Information. The single-cell image data for model building are available through the figshare website with the download link of 10.6084/m9.figshare.13070666.v1.  Source data are provided with this paper.

## Source data

Source Data

## Code availability

The code for model building is available through the link 10.5281/zenodo.4606918
