# Supplementary material for: Deep learning-based predictive identification of neural stem cell differentiation
Source: Nat Commun. 2021 May 10;12:2614. doi: 10.1038/s41467-021-22758-0 (PMC8110743; doi:10.1038/s41467-021-22758-0)
Supplement: Supplementary file 1 — Supplementary Information [file 41467_2021_22758_MOESM1_ESM.pdf]

Deep learning-based predictive identification of neural stem cell differentiation

Yanjing Zhu[1#], Ruiqi Huang[1#], Zhourui Wu[1], Simin Song[1], Liming Cheng[1*], Rongrong Zhu[1*]

1 Key Laboratory of Spine and Spinal Cord Injury Repair and Regeneration of Ministry of Education, Department of Orthopaedics, Tongji Hospital, Tongji University School of Medicine, School of Life Science and Technology, Tongji University, Shanghai, P. R. China

# These authors contributed equally to this work.

*Corresponding authors:

Liming Cheng*, Tel: 862166111283; Fax:862156050502; E-mail: limingcheng@tongji.edu.cn

Affiliation: Key Laboratory of Spine and Spinal Cord Injury Repair and Regeneration of Ministry of Education, Department of Orthopedics, Tongji Hospital, Tongji University School of Medicine, School of Life Science and Technology, Tongji University, Shanghai 200065, China

Rongrong Zhu*, Tel: 862166111283; Fax:862156050502; E-mail: rrzhu@tongji.edu.cn

Affiliation: Key Laboratory of Spine and Spinal Cord Injury Repair and Regeneration of Ministry of Education, Department of Orthopedics, Tongji Hospital, Tongji University School of Medicine, School of Life Science and Technology, Tongji University, Shanghai 200065, China

Supplementary information

Supplementary Tables

Supplementary Table 1. NeuN/GFAP/Olig2 positive cell proportion (%) of each NSCs differentiation group by immunofluorescence counting and brightfield deep neural network model benchmarking.

	**Count**	**Benchmark**	**Count**	**Benchmark**	**Count**	**Benchmark**
NeuN positive ratio (NSCs differentiate into neurons)						
Duration / Inducer	1 day		3 days		5 days	
Neuron differentiation medium (RA)	55.8267	82.72892	67.4267	87.94307	82.0733	82.97511
NT3	20.9967	73.67773	55.2933	72.48147	75.4067	74.2915
LDH-NT3	48.73	71.89459	60.06	72.09604	68.8233	74.29351
CNTF	25.3667	76.92308	34.23	75.02851	78.3333	79.59893
NGF	33.0333	81.09294	33.4667	83.66849	82.6333	81.97306
NT4	10.4333	74.197	29.2333	72.29602	71.3333	75.78475
MT	35.6333	76.79027	55.7667	78.32898	79.2667	80.28657
GFAP positive ratio (NSCs differentiate into astrocytes)						
Duration / Inducer	0.5 day		1 day		2 days	
Astrocyte differentiation medium	83.3567	95.86375	83.8133	98.99422	88.78	97.45879
Olig2 positive ratio (NSCs differentiate into oligodendrocytes)						
Duration / Inducer	1 day		2 days		3 days	
Oligodendrocyte differentiation medium	52.9679	80.59837	55.4719	74.68355	63.6218	92.05038

Supplementary Table 2. Accuracy (%) of NSCs differentiation identification at various conditions on both darkfield and brightfield models.

	**Darkfield**	**Brightfield**	**Darkfield**	**Brightfield**	**Darkfield**	**Brightfield**
Duration / Inducer	1 day		3 days		5 days	
Neuron differentiation medium (RA)	98.05077	82.72892	100	87.94306	100	82.97511
NT3	100	73.67773	100	72.48147	100	74.2915
LDH-NT3	100	71.89459	100	72.09604	100	74.29351
Duration / Inducer	0.5 day		1 day		2 days	
Astrocyte differentiation medium	99.22141	95.86375	99.95721	98.99422	100	97.45879
Duration / Inducer	0.5 day		1 day		2 days	
Oligodendrocyte differentiation medium	100	80.59837	100	74.68355	100	92.05038

Supplementary Table 3. Primer sequences for real-time PCR of target genes.

Primer name	Primer sequence
GAPDH	Forward Primer: 5' –AGTGCCAGCCTCGTCTCATA– 3'<br>Reverse Primer: 5' –TGAACTTGCCGTGGGTAGAG– 3'
Nestin	Forward Primer: 5' –TGGAGCAGGAGAAGCAAGGTCTAC – 3'<br>Reverse Primer: 5' –GGACATCTTGAGGTGTGCCAGTTG– 3'
NeuN	Forward Primer: 5' –GCACAGACAGATAGCCAGCA– 3'<br>Reverse Primer: 5' –TCCCGAATTGCCCGAACATT– 3'
Tuj1	Forward Primer: 5' –GGCAACTATGTGGGGGACTC– 3'<br>Reverse Primer: 5' –GCACCACTCTGACCGAAGATA– 3'
GFAP	Forward Primer: 5' –GAGTTACCAGGAGGCACTCG– 3'<br>Reverse Primer: 5' –TACAGGAATGGTGATGCGGT– 3'
Olig2	Forward Primer: 5' –TCAAGTCATCTTCCTCCAGCAC– 3'<br>Reverse Primer: 5' –GGCTCAGTCATCTGCTTCTTATCTT– 3'

Supplementary Figures

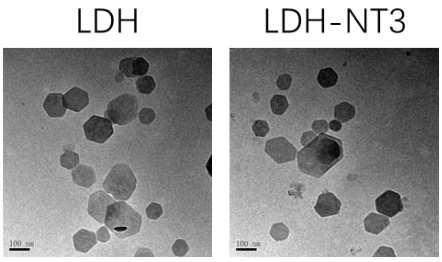

Supplementary Figure 1. TEM images of LDH and LDH-NT3 (scale bar=100nm). LDH is abbreviation of layered double hydroxide.

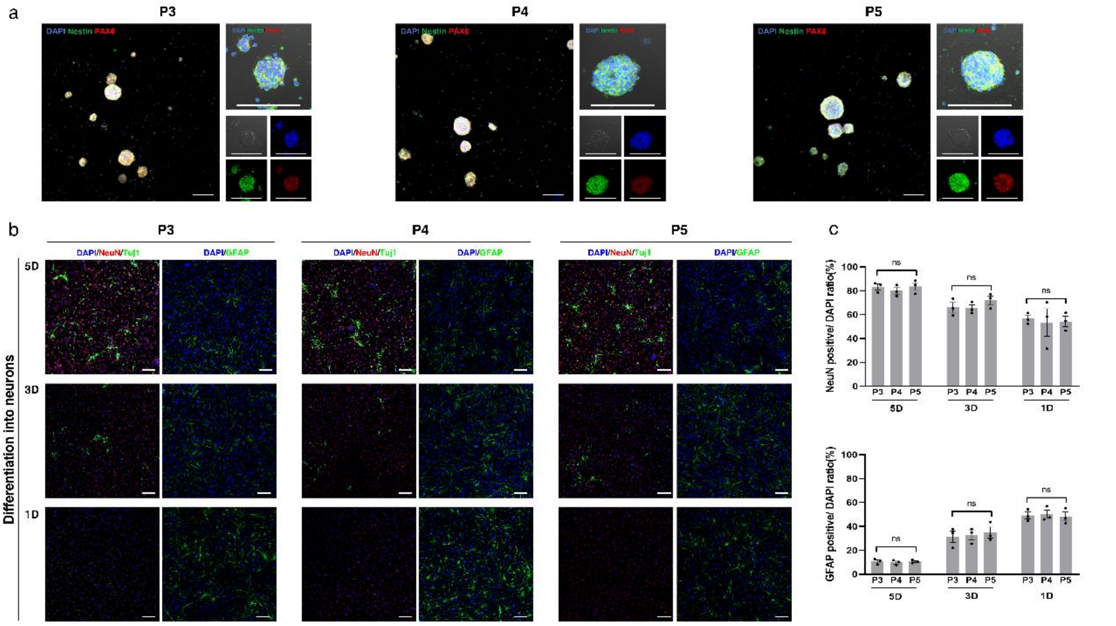

Supplementary Figure 2. **Determination of proliferation and differentiation abilities of NSCs at p3-p5. a**, Images of immunofluorescent staining using Nestin and PAX6 at 5D (5 days), 3D (3 days) and 1D (1 day). **b**, Immunofluorescent staining images of RA (retinoic acid) -treated (neuron differentiation induction) NSCs at p3-p5 using NeuN, Tuj1 and GFAP. **c**, Quantification of immunostaining data. The Y axis shows the numbers of NeuN and GFAP positive cells. Data are shown as mean ± SEM, n=3 imaging fields repeats. Statistical significance was determined by two-sided Welch's ANOVA. ns: not significant. Scale bar=100 μm.

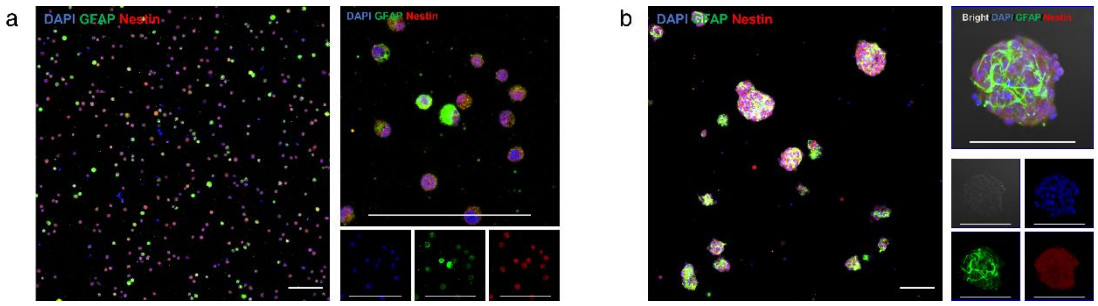

Supplementary Figure 3. Immunofluorescence images of **a**, Single NSC and **b**, neural spheres express Nestin and GFAP. Scale bar=100 μm.

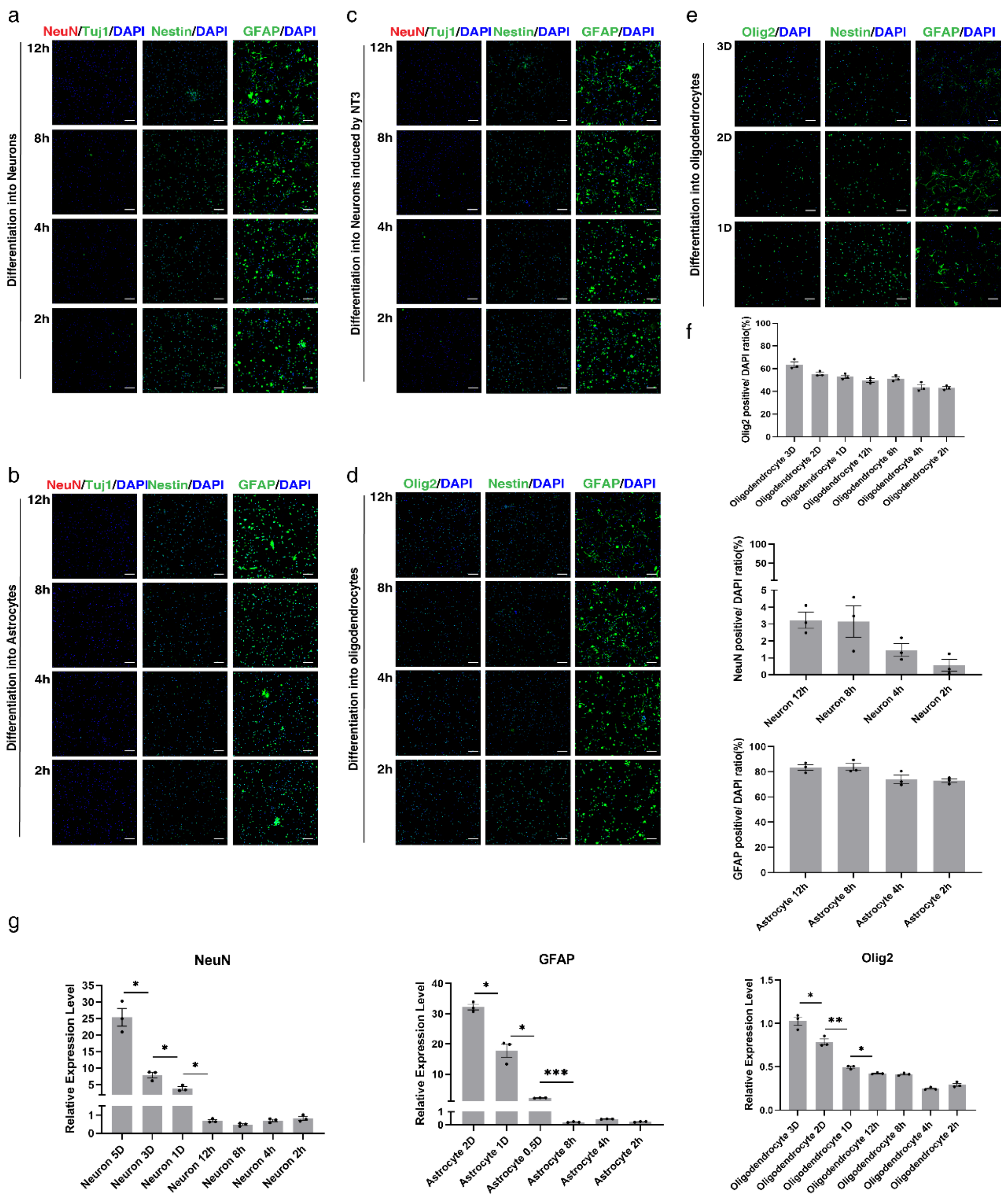

Supplementary Figure 4. **Expressions of specific markers in differentiated NSCs identified by immunofluorescence and RT-qPCR.** Images of immunofluorescent staining using NeuN, Tuj1, GFAP, Olig2 and Nestin as characteristic markers of **a**, neurons (RA-treated), **b**, astrocytes, **c,** neurons (NT3-treated), and using Olig2, GFAP and Nestin as characteristic markers of **d and e,** oligodendrocyte differentiation induction at 3D (3 days), 2D (2 days), 1D (1 day), 0.5D (0.5 days), 12h (12 hours), 8h (8 hours), 4h (4 hours) and 2h (2 hours). **f,** Quantification of immunostaining data, n=3 imaging fields repeats. **g**, Quantitative real-time PCR detection of NeuN, GFAP and Olig2 gene expression, n=3 biological repeats. Data are shown as mean ± SEM. Statistical significance was determined by two-sided Welch's ANOVA. *P<0.05, **P<0.01, ***P<0.001. Scale bar=100 μm.

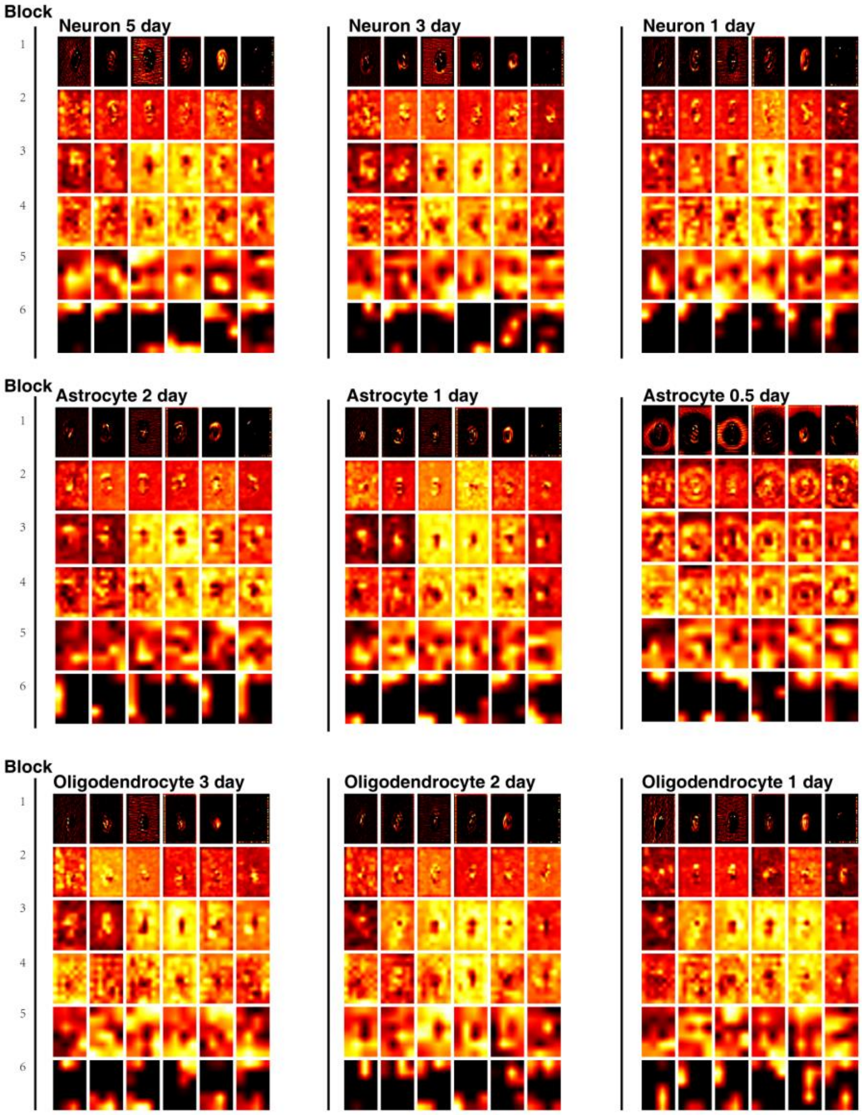

Supplementary Figure 5. Activation map examples of each layer blocks of the model for cell images randomly selected from each treated group.

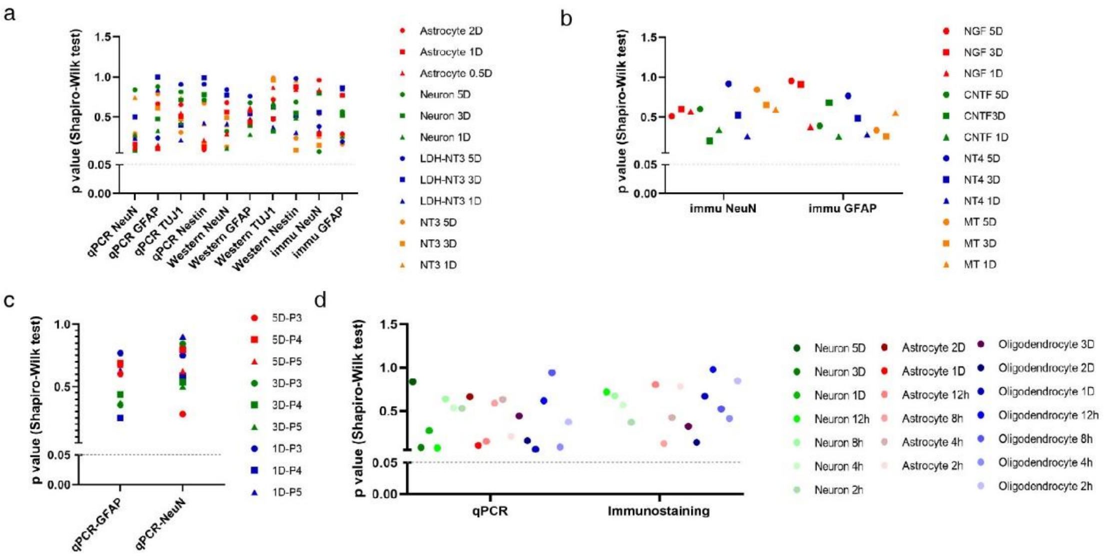

Supplementary Figure 6. Normality test for Fig. 2 (**a**), Fig. 3 (**b**), Supplementary Fig. 2 (**c**) and Supplementary Fig. 4 (**d**) using two-sided Shapiro-Wilk test. The Y axis shows P value, $P > 0.05$ was taken as for passing normality test.
