# Supplementary material for: Deep learning-based predictive identification of neural stem cell differentiation
Source: Nat Commun. 2021 May 10;12:2614. doi: 10.1038/s41467-021-22758-0 (PMC8110743; doi:10.1038/s41467-021-22758-0)
Supplement: Supplementary file 2 — Reporting Summary [file 41467_2021_22758_MOESM2_ESM.pdf]

nature research

Corresponding author(s): Rongrong Zhu

Last updated by author(s): Mar 23, 2021

Reporting Summary

Nature Research wishes to improve the reproducibility of the work that we publish. This form provides structure for consistency and transparency in reporting. For further information on Nature Research policies, see our Editorial Policies and the Editorial Policy Checklist.

Statistics

For all statistical analyses, confirm that the following items are present in the figure legend, table legend, main text, or Methods section.

n/a	Confirmed	
☐	☒	The exact sample size (*n*) for each experimental group/condition, given as a discrete number and unit of measurement
☐	☒	A statement on whether measurements were taken from distinct samples or whether the same sample was measured repeatedly
☐	☒	The statistical test(s) used AND whether they are one- or two-sided<br>*Only common tests should be described solely by name; describe more complex techniques in the Methods section.*
☒	☐	A description of all covariates tested
☐	☒	A description of any assumptions or corrections, such as tests of normality and adjustment for multiple comparisons
☐	☒	A full description of the statistical parameters including central tendency (e.g. means) or other basic estimates (e.g. regression coefficient) AND variation (e.g. standard deviation) or associated estimates of uncertainty (e.g. confidence intervals)
☐	☒	For null hypothesis testing, the test statistic (e.g. *F*, *t*, *r*) with confidence intervals, effect sizes, degrees of freedom and *P* value noted<br>*Give P values as exact values whenever suitable.*
☒	☐	For Bayesian analysis, information on the choice of priors and Markov chain Monte Carlo settings
☒	☐	For hierarchical and complex designs, identification of the appropriate level for tests and full reporting of outcomes
☒	☐	Estimates of effect sizes (e.g. Cohen's *d*, Pearson's *r*), indicating how they were calculated

Our web collection on statistics for biologists contains articles on many of the points above.

Software and code

Policy information about availability of computer code

Data collection	ImageStream Mark II apparatus (Merck Millipore), FlowSight apparatus (Merck Millipore), IDES v6.1, Zeiss confocal microscope (LSM 700, Carl Zeiss, Jena, Germany), ZEN 2.3 (blue edition, Carl Zeiss), ImageQuant LAS 4000 mini (GE Healthcare Life Science), Nanodrop ND-2000 (Thermo Science, MA, USA), LightCycler Real-Time PCR System (Roche, 480II).
Data analysis	Image-J 1.47v, Microsoft Excel version 16.36, Adobe Illustrator CC 2018, GraphPad Prism 8 version 8.4.3 were used. The code for this study is available through the link of https://doi.org/10.5281/zenodo.4606918

For manuscripts utilizing custom algorithms or software that are central to the research but not yet described in published literature, software must be made available to editors and reviewers. We strongly encourage code deposition in a community repository (e.g. GitHub). See the Nature Research guidelines for submitting code & software for further information.

Data

Policy information about availability of data

All manuscripts must include a data availability statement. This statement should provide the following information, where applicable:

- Accession codes, unique identifiers, or web links for publicly available datasets
- A list of figures that have associated raw data
- A description of any restrictions on data availability

The main data supporting the findings of this study are available within the article and its Supplementary Information. The single cell image data for model building is available through the figshare website with the download link of https://doi.org/10.6084/m9.figshare.13070666.v1. The raw numbers for charts and graphs (corresponding data points), specific data p values and unprocessed scans of Western blot are available in the Source Data file whenever possible. Source data are provided with this paper.

Field-specific reporting

Please select the one below that is the best fit for your research. If you are not sure, read the appropriate sections before making your selection.

- [x] Life sciences
- [ ] Behavioural & social sciences
- [ ] Ecological, evolutionary & environmental sciences

For a reference copy of the document with all sections, see nature.com/documents/nr-reporting-summary-flat.pdf

Life sciences study design

All studies must disclose on these points even when the disclosure is negative.

Sample size	No statistical measures were used to determine sample size. Sample sizes were estimated based on prior experiments in our laboratory and previously published data in reference to Nature. 2012 November 22; 491(7425): 603–607. doi:10.1038/nature11557.
Data exclusions	No data was excluded
Replication	All experimental findings could reliably be reproduced independently three times.
Randomization	For immunostaining, flow cytometry, Western Blot and RT-qPCR experiments, cultured cells groups were set randomly. Imaging fields were stochastically selected during image acquisition. For model building, single cell images were randomly divided into training and testing groups.
Blinding	All experiments were not performed blind. Each experiment was designed with proper controls, and samples for comparison were collected and analyzed under the same conditions. However, the investigators were blinded to group allocation during data collection, and the persons performing data analysis were unaware of the sample identity.

Reporting for specific materials, systems and methods

We require information from authors about some types of materials, experimental systems and methods used in many studies. Here, indicate whether each material, system or method listed is relevant to your study. If you are not sure if a list item applies to your research, read the appropriate section before selecting a response.

Materials & experimental systems

n/a	Involved in the study	
☐	☒	Antibodies
☐	☒	Eukaryotic cell lines
☒	☐	Palaeontology and archaeology
☐	☒	Animals and other organisms
☒	☐	Human research participants
☒	☐	Clinical data
☒	☐	Dual use research of concern

Methods

n/a	Involved in the study	
☒	☐	ChIP-seq
☐	☒	Flow cytometry
☒	☐	MRI-based neuroimaging

Antibodies

Antibodies used	Antibodies used for immunostaining includes: anti-NeuN (Abcam, catalogue no. ab190195, conjugated with Alexa Fluor 488, 1:200), NeuN (Abcam, catalogue no. ab104225, 1:500), anti-NeuN (Millipore, catalogue no. MAB 377, 1:100), anti-GFAP (Abcam, catalogue no. ab53554, 1:1000), anti-Tuj1 (Abcam, catalogue no. ab78078, 1:1000), anti-Nestin (Abcam, catalogue no. ab6320, 1:500), anti-PAX6 (Abcam, catalogue no. ab195045, 1:350), anti-Olig2 (Abcam, catalogue no. ab109186, 1:100), rabbit anti-mouse IgG (YEASEN, catalogue no. 33912ES60, 1:200), donkey anti-mouse IgG (Abcam, catalogue no. ab150105, 1:1000), donkey anti-goat IgG (Abcam, catalogue no. ab175704, 1:1000), and donkey anti-rabbit IgG (Abcam, catalogue no. ab150075, 1:1000).<br>Antibodies used for western blots include: anti-NeuN (Abcam, catalogue no. ab104225, 1:5000), anti-Nestin (Abcam, catalogue no. ab6320, 1:1000), anti-GFAP (Abcam, catalogue no. ab53554, 1:10000), anti-Tuj1 (Abcam, catalogue no. ab78078, 1:1000), anti-β-actin (ABWAYS, catalogue no. AB0035, 1:5000), goat anti-mouse HRP (Abcam, catalogue no. ab205719, 1:5000), and goat anti-rabbit HRP (Abcam, catalogue no. ab205718, 1:5000).<br>Antibodies used for flow cytometry include: anti-GFAP (Bioss, catalogue no. bs-0199R-AF488, 1:200), anti-NeuN (Bioss, catalogue no. bs-10394R-APC, 1:200) and anti-Olig2 (Bioss, catalogue no. bs-11194R-PE, 1:200)
Validation	All antibodies have been validated by companies with suitable controls, and reported by previous studies. The validation statements including relevant citations and antibody profile can be found on the websites of manufacturers which are showed as below.<br>-anti-NeuN (Abcam, catalogue no. ab190195, conjugated with Alexa Fluor 488),<br>https://www.abcam.com/alexa-fluor-488-neun-antibody-epr12763-neuronal-marker-ab190195.html<br>-anti-NeuN (Abcam, catalogue no. ab104225),<br>https://www.abcam.com/neun-antibody-neuronal-marker-ab104225.html<br>-anti-NeuN (Millipore, catalogue no. MAB 377),

https://www.merckmillipore.com/JP/ja/product/Anti-NeuN-Antibody-clone-A60,MM_NF-MAB377?ReferrerURL=https%3A%2F%2Fcn.bing.com%2F&bd=1
-anti-GFAP (Abcam, catalogue no. ab53554),
https://www.abcam.com/gfap-antibody-ab53554.html
-anti-Tuj1 (Abcam, catalogue no. 78078),
https://www.abcam.com/beta-iii-tubulin-antibody-2g10-neuronal-marker-ab78078.html
-anti-Nestin (Abcam, catalogue no. ab6320),
https://www.abcam.com/nestin-antibody-196908-ab6320.html
-anti-PAX6 (Abcam, catalogue no. ab195045),
https://www.abcam.com/pax6-antibody-epr15858-ab195045.html
-anti-Olig2 (Abcam, catalogue no. ab109186),
https://www.abcam.com/olig2-antibody-epr2673-ab109186.html
-rabbit anti-mouse IgG (YEASEN, catalogue no. 33912ES60),
https://www.yeasen.com/products/detail/1625
-donkey anti-mouse IgG (Abcam, catalogue no. ab150105),
https://www.abcam.com/donkey-mouse-igg-hl-alexa-fluor-488-ab150105.html
-donkey anti-goat IgG (Abcam, catalogue no. ab175704),
https://www.abcam.com/donkey-goat-igg-hl-alexa-fluor-568-preadsorbed-ab175704.html
-and donkey anti-rabbit IgG (Abcam, catalogue no. ab150075),
https://www.abcam.com/donkey-rabbit-igg-hl-alexa-fluor-647-ab150075.html
-anti-GFAP (Bioss, catalogue no. bs-0199R-AF488),
http://www.bioss.com.cn/prolook_03.asp?id=AF08169606000384&pro37=1
-anti-NeuN (Bioss, catalogue no. bs-10394R-APC),
http://www.bioss.com.cn/prolook_03.asp?id=AF08169606018453&pro37=1
-anti-Olig2 (Bioss, catalogue no. bs-11194R-PE),
http://www.bioss.com.cn/prolook_03.asp?id=AF08169606014494&pro37=1
-anti-β-actin (ABWAYS, catalogue no. AB0035),
http://abways.com/showproduct.asp?cid=AB0035
-goat anti-mouse HRP (Abcam, catalogue no. ab205719),
https://www.abcam.com/goat-mouse-igg-hl-hrp-ab205719.html
-goat anti-rabbit HRP (Abcam, catalogue no. ab205718),
https://www.abcam.com/goat-rabbit-igg-hl-hrp-ab205718.html

Eukaryotic cell lines

Policy information about cell lines

Cell line source(s)	Embryonic SD rats (E13.5) were chosen for cell extraction.
Authentication	The cell lines used were checked for morphology by microscopy and immunostaining with specific markers.
Mycoplasma contamination	We periodically checked potential contamination with mycoplasma, which causes retarded cell growth and low pH in the media. All cell lines tested negative for mycoplasma contamination by Hoechst staining of the cells.
Commonly misidentified lines (See ICLAC register)	No. The cell lines used are not listed in the database.

Animals and other organisms

Policy information about studies involving animals; ARRIVE guidelines recommended for reporting animal research

Laboratory animals	Embryonic SD rats (E13.5)
Wild animals	No wild animals were involved.
Field-collected samples	No field-collected animals were involved.
Ethics oversight	All protocols were approved by the Institute of Laboratory Animal Resources of Tongji University and complied with the Guide for the Care and Use of Laboratory Animals of the National Institutes of Health. Ethical and legal approval for this study was obtained from the Institute of Laboratory Animal Resources Animal Care and Use Committee.

Note that full information on the approval of the study protocol must also be provided in the manuscript.

Flow Cytometry

Plots

Confirm that:

☐ The axis labels state the marker and fluorochrome used (e.g. CD4-FITC).

☐ The axis scales are clearly visible. Include numbers along axes only for bottom left plot of group (a 'group' is an analysis of identical markers).

☐ All plots are contour plots with outliers or pseudocolor plots.

☐ A numerical value for number of cells or percentage (with statistics) is provided.

Methodology

Sample preparation	NSCs treated with differentiation medium were collected with Accutase at different stages and washed thrice with PBS before being fixed. The cells were fixed in cold 80% methanol for 5 minutes and then disrupted using 0.1% Triton X-100 on ice for 20 minutes. Later, the cells were incubated with anti-GFAP (Bioss, catalogue no. bs-0199R-AF488, 1:200), anti-NeuN (Bioss, catalogue no. bs-10394R-APC, 1:200) and anti-Olig2 (Bioss, catalogue no. bs-11194R-PE, 1:200) antibodies for 2 hours on ice. The cells were washed three times with PBS before flow cytometry.
Instrument	FlowSight apparatus (Merck Millipore) , ImageStream Mark II apparatus (Merck Millipore)
Software	IDEAS v6.1 was used for image data exploration and analysis.
Cell population abundance	Approximately 5000-20,000 stained cells.
Gating strategy	Visual Flowsight data were used to gain single cell images. gating strategy, plots and statistic analyze were nor involved.

☐ Tick this box to confirm that a figure exemplifying the gating strategy is provided in the Supplementary Information.
